# Molar loss induces hypothalamic and hippocampal astrogliosis in aged mice

**DOI:** 10.1038/s41598-022-10321-w

**Published:** 2022-04-18

**Authors:** Masae Furukawa, Hirobumi Tada, Jingshu Wang, Mitsuyoshi Yamada, Mie Kurosawa, Akiko Satoh, Noboru Ogiso, Yosuke Shikama, Kenji Matsushita

**Affiliations:** 1 Department of Oral Disease Research, Geroscience Research Center, National Center for Geriatrics and Gerontology, Obu, Japan; 2 Department of Nutrition, Faculty of Wellness, Shigakkan University, Obu, Japan; 3 Department of Inflammation and Immunosenescence, Geroscience Research Center, National Center for Geriatrics and Gerontology, Obu, Japan; 4 Department of Operative Dentistry, School of Dentistry, Aichi Gakuin University, Nagoya, Japan; 5 Department of Integrative Physiology, Geroscience Research Center, National Center for Geriatrics and Gerontology, Obu, Japan; 6 Department of Integrative Physiology, Institute of Development, Aging, and Cancer, Tohoku University, Sendai, Japan; 7 Department of Laboratory of Experimental Animals, National Center for Geriatrics and Gerontology, Obu, Japan

**Keywords:** Neuroscience, Cognitive ageing, Cognitive neuroscience, Learning and memory, Neural ageing

## Abstract

Age-related tooth loss impedes mastication. Epidemiological and physiological studies have reported that poor oral hygiene and occlusion are associated with cognitive decline. In the present study, we analyzed the mechanism by which decreased occlusal support following bilateral extraction of the maxillary first molars affects cognitive functions in young and aged mice and examined the expression of brain-function-related genes in the hippocampus and hypothalamus. We observed decreased working memory, enhanced restlessness, and increased nocturnal activity in aged mice with molar extraction compared with that in mice with intact molars. Furthermore, in the hypothalamus and hippocampus of molar-extracted aged mice, the transcript-level expression of *Bdnf*, *Rbfox3*, and *Fos* decreased, while that of *Cdkn2a* and *Aif1* increased. Thus, decreased occlusal support after maxillary first molar extraction may affect cognitive function and activity in mice by influencing aging, neural activity, and neuroinflammation in the hippocampus and hypothalamus.

## Introduction

Dental health has been selected by the World Health Organization (WHO) as one of the ten criteria that should be met to maintain human health^1^. Number of teeth present is an indicator of a person’s oral health^2^. Teeth are not only crucial for mastication, but they also play an essential role in determining overall nutrition and general health^2^. Tooth loss affects the quality of life^3^, and has a profound impact on aesthetics, nutrition, and dietary choices^4^. Aging, poor oral care, and injury may lead to tooth loss. Tooth loss in adults is also associated with increased risk of obesity, diabetes, cardiovascular diseases, and certain types of cancer^5^. As the incidence of these systemic diseases increases with age, maintaining one’s tooth count and health have become increasingly important for an aging society.

Chewing not only enables the mechanical crushing of food and aids digestion but also stimulates the central nervous system, which increases the temperature in the brain, improves cerebral blood flow, and activates the metabolism in the brain; this in turn, stimulates the nervous system and contributes to homeostasis^6^. Tooth loss is a risk factor for various forms of dementia^7^, including Alzheimer’s disease^8^, and other causes of cognitive decline, such as Parkinson's disease^9,10^.

Some studies have analyzed the effects of tooth extraction and surviving on liquid diets on the decline of learning, cognitive, and memory functions in mouse and rat models on the basis of behavioral physiology^11,12^, but there has been no detailed analysis of the changes in molecular expression in the brain associated with these alterations. However, the association between tooth loss and the molecular pathogenesis underlying hypothalamic and hippocampal senescence is still unknown. Thus far, clinical studies have shown that tooth loss affects cognitive function and causes sleep disturbance^13^, and a few studies have examined the mouse hippocampus and hypothalamus in depth under the same rearing environment^14,15^. These studies highlighted the importance of the hypothalamus and hippocampus as areas of the brain related to cognitive functions.

Therefore, we aimed to elucidate the age-related changes in the hippocampus and hypothalamus. In this study, we tested the hypothesis that hypothalamic and hippocampal senescence is enhanced by molar loss.

## Results

### Changes in weight and stress levels due to the loss of a maxillary first molar

**Figure 1:**
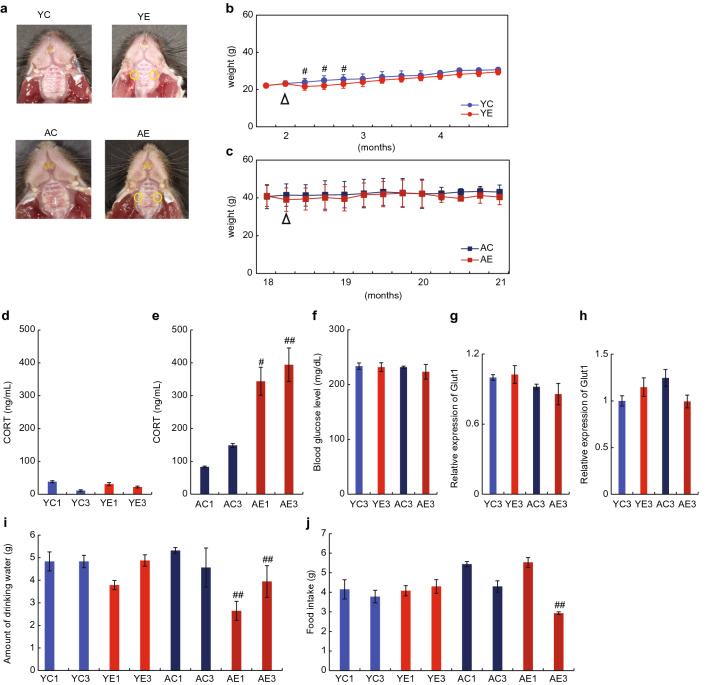
Changes in the weight and stress levels in response to the loss of a maxillary first molar. (**a**) Intraoral photographs of mice after molar extraction. The circled area indicates the extraction socket (1 month after extraction). *YC* young control group, *YE* young extraction group, *AC* aged control group, *AE* aged extraction group. (**b**) Weight changes in the young group. The vertical axis indicates weight, and the horizontal axis indicates the number of months. The triangular portion indicates the time of molar extraction. (**c**) Weight changes in the aged groups. (**d**) Urinary corticosterone levels in mice from the YC and YE groups (ng/mL). E1 indicates 1 month after molar extraction, and E3 indicates 3 months after molar extraction. (**e**) Urinary corticosterone levels in mice from the AC and AE groups (ng/mL). (**f**) Blood glucose level (mg/dL). (**g**) Expression of *Glut1* in the hypothalamus. (**h**) Expression of *Glut1* in the hippocampus. Results are presented as means ± SE. YC(E)3 represent young mice groups 3 months after tooth extraction, respectively. The teeth of aged mice were extracted at 18 months of age, AC(E)3 indicates 3 months (21 M) after tooth extraction. (**i**) Amount of drinking water per day (g). (**j**) Amount of food intake per day (g). ^##^P < 0.01; ^#^P < 0.05 vs. control (Tukey’s post-hoc test). ^##^P < 0.01; ^#^P < 0.05 vs. control (*t*-test). Results are presented as means ± SE.

To investigate the effects of maxillary first molar loss on memory and learning functions, we extracted the maxillary first molars bilaterally of 6-week-old (young) and 18-month-old (aged) mice (Fig. 1a). We observed changes in the physical conditions of the mice over the next 3 months. Although no change was observed in the first month post-extraction, a significant decrease (P < 0.012) in body weight was observed in young mice that had their molar teeth extracted (compared with that of young mice with intact molars). However, after the 1-month time point, no significant difference in body weight was observed between the young mice with and without their molars extracted, and their body weights increased over time (Fig. 1b). In contrast, no significant change in weight was observed in aged mice with molars extracted for 3 months after tooth extraction (AE and AC, Fig. 1c). Next, we examined the presence of tooth-loss-related stress in the mice after tooth extraction using urinary corticosterone levels as an indicator. Urinary corticosterone levels in the YC1 and YE1 groups were 38.4 ± 3.0 ng/mL and 31.1 ± 4.5 ng/mL, respectively (P = 0.26) (Fig. 1d). In the YC3 and YE3 groups, these values were 11.06 ± 2.7 ng/mL and 22.3 ± 2.7 ng/mL, respectively (P = 0.23). However, the urinary concentration in aged mice in the AC1 group (83.2 ± 2.5 ng/mL) was substantially lower than that in mice in the AE1 group (343.95 ± 42.6 ng/mL) (P < 0.025). In the AC3 and AE3 groups, these values were 148.5 ± 6.0 ng/mL and 394.1 ± 51.2 ng/mL, respectively (P < 0.0081) (Fig. 1e).

Next, we measured blood glucose levels 3 months after tooth extraction. In the YC3 and YE3 groups, these values were 233.67 ± 5.78 mg/dL and 231.67 ± 8.11 mg/dL, respectively (P = 0.85). In the AC3 and AE3 groups, these values were 232.67 ± 1.86 mg/dL and 223.34 ± 13.4 mg/dL, respectively (P = 0.57). Results showed no change in blood glucose levels in either young or aged mice after molar extraction (Fig. 1f).

We examined the mRNA expressions of *Glut1*. The results revealed that the expression of *Glut1* mRNA in the hypothalamus and hippocampus remained unchanged in both the YE3 and AE3 groups (Fig. 1g,h).

In addition, we evaluated the amount of feed and water intake after tooth extraction (Fig. 1i). In young mice, the amount of water (YC1: 4.83 ± 0.4 g) consumed decreased slightly 1 month after tooth extraction (YE1: 3.78 ± 0.2 g), but the difference was not significant. There was no difference in the amount of water consumed between the YC3 (4.83 ± 0.3 g) and YE3 (4.88 ± 0.3 g) groups.

In young mice, the amount of feed intake did not change after tooth extraction (Fig. 1j). However, in aged mice, the amount of both water and feed intake decreased significantly 3 months after tooth loss. Specifically, the water intake amounts were: AC1 (5.32 ± 0.1 g) and AE1 (2.6 ± 0.4 g), and AC3 (4.6 ± 0.9 g) and AE3 (3.9 ± 0.7 g); and the feed intake amounts were: AC1 (5.44 ± 0.1 g) and AE1 (5.53 ± 0.3 g), and AC3 (4.3 ± 0.3 g) and AE3 (2.93 ± 0.1 g) (Fig. 1f,g).

### Effects of maxillary molar loss on mouse memory and behavior

**Figure 2:**
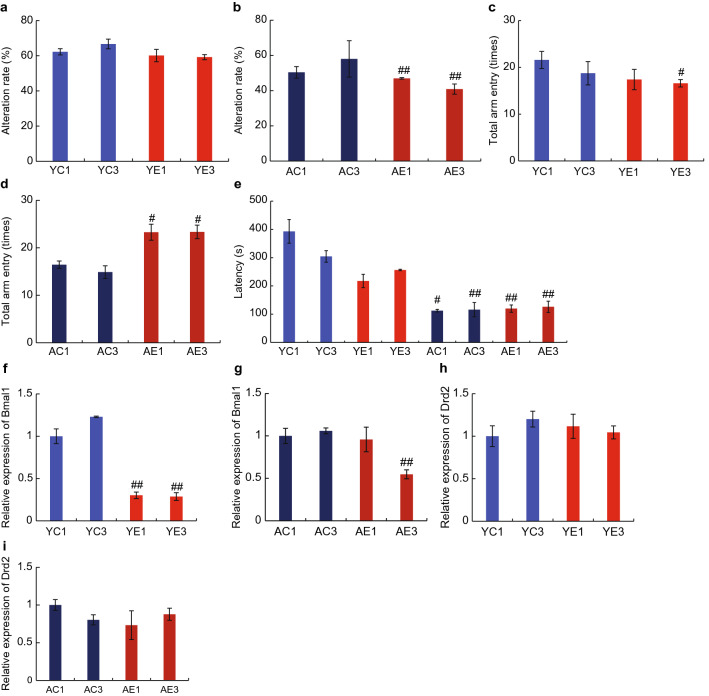
Effects of maxillary molar loss on mouse memory and behavior. (**a**) The alteration rate in young mice in the Y-maze experiment. (**b**) The alteration rate in aged mice in the Y-maze experiment. (**c**) Total arm entry in the young group in the Y-maze experiment. (**d**) Total arm entry in aged mice in the Y-maze experiment. (**e)** Latency in young and aged mice in the rotarod experiment. (**f,g**) Expression of *Bmal1* in the hypothalamus. (**h,i**) Expression of *Drd2* in the hypothalamus. ^##^P < 0.01; ^#^P < 0.05 vs. control (Tukey’s post-hoc test). Results are presented as means ± SE. YC(E)1, YC(E)2, and YC(E)3 represent young mice groups immediately after tooth extraction, 1 month after, and 3 months after tooth extraction, respectively. The teeth of aged mice were extracted at 18 months of age, AC(E)1 indicates mice aged 18 months, AC(E)2 indicates 1 month after (19 M), and AC(E)3 indicates 3 months (21 M) after tooth extraction.

We used the Y-maze test to evaluate the mechanism by which the loss of maxillary molars affects the learning and memory abilities of mice. We first performed a spontaneous alternation test to evaluate the spatial working memory of the mice. The alternation rates in the YE1 and YE3 groups were not significantly different from those in the YC1 and YC3 groups, respectively (Fig. 2a). However, the alternation rates decreased by approximately 20% in the AE1 and AE3 groups, compared with those in the AC1 and AC3 groups (Fig. 2b). These results suggest that working memory was negatively affected by molar extraction in aged mice.

Next, we investigated changes in the spontaneous movement of tooth-extracted mice using the number of arm entries in the Y-maze as an index. In young mice, tooth extraction tended to reduce the number of arm entries (Fig. 2c). Meanwhile, in the AE1 and AE3 groups, the number of arm entries significantly increased compared with those in the AC1 and AC3 groups, respectively (Fig. 2d) (AC1 versus AE1: P = 0.011; AC3 versus AE3: P = 0.01).

The effects of tooth loss on motor learning functions were examined using the rotor-rod test. The young mice stayed on the rods for a significantly longer period of time than the aged mice (AC1 versus YC: P = 0.016; AC3 versus YC: P = 0.009; AE1 versus YC: P = 0.005; AE3 versus YC: P = 0.003) (Fig. 2e). The latency time in the rotor-rod test was significantly shorter for the YE1 group than the YC1 group (Fig. 2e), whereas in the YE3 group, the latency time recovered to the same level as that of the YC3 group. No change in latency time was observed in response to tooth extraction in aged mice (Fig. 2e). To investigate further, we also examined gene expression changes in the hypothalamus of the extraction and control group mice. We examined the mRNA expressions of brain and muscle Arnt-like 1 (*Bmal1*) and *Drd2*. The results revealed that the expression of *Bmal1* mRNA in the hypothalamus was significantly decreased in both the YE and AE3 groups (Fig. 2f,g), while that of *Drd2* mRNA remained unchanged (Fig. 2h,i).

### Effects of maxillary molar loss on molecular expression in the hippocampus of mice

The loss of molars is associated with a decline in cognitive function in mice^14,15^. To identify the molecular changes in the brain in response to tooth removal, gene expression in the hippocampus was assessed using real-time PCR.

**Figure 3:**
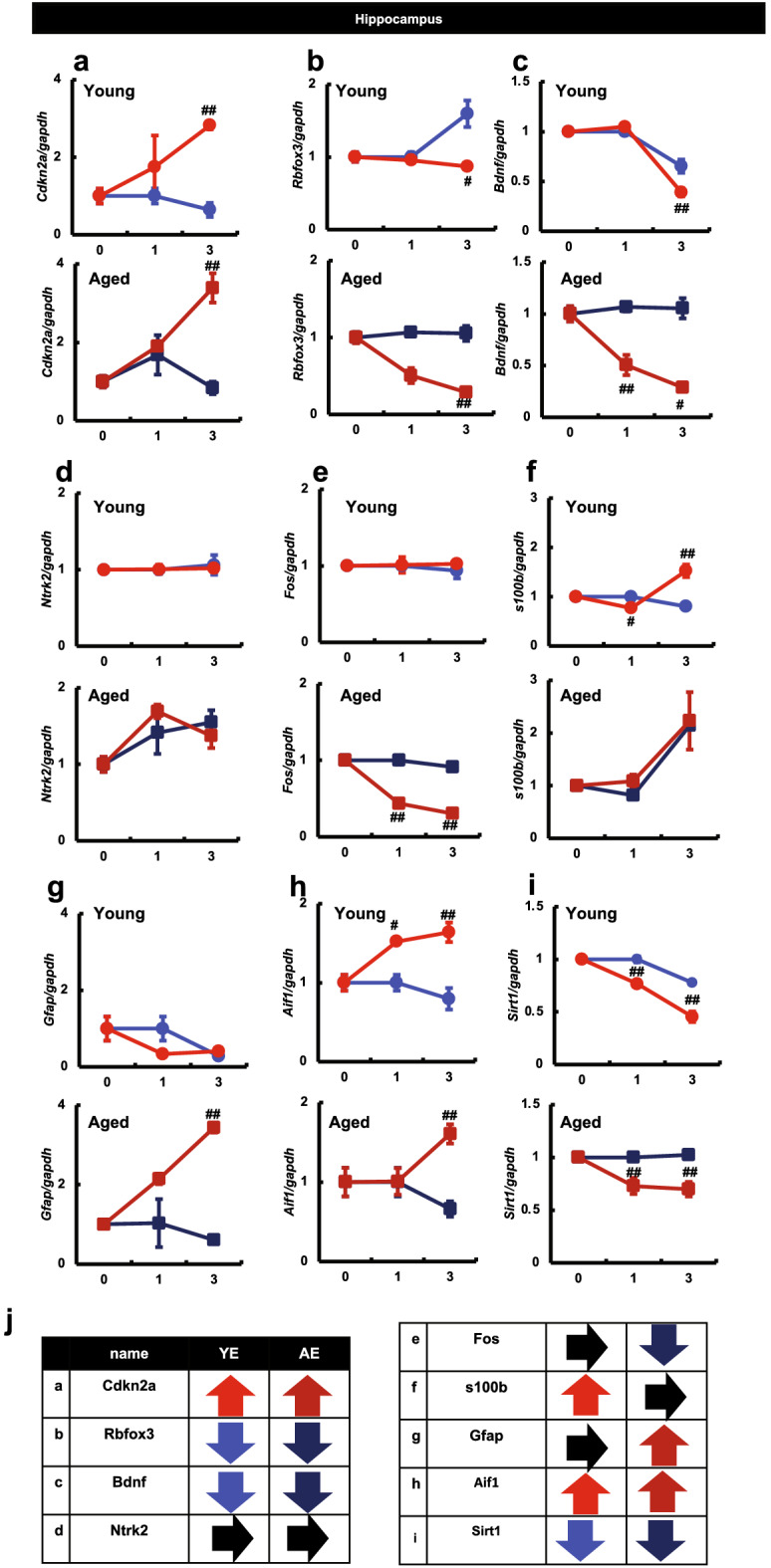
Effects of maxillary molar loss on gene expression in the hippocampus of mice (Real-time PCR). The vertical axis indicates the expression level. The horizontal axis indicates the month, with YC1 or AC1 as 1. ^##^P < 0.01; ^#^P < 0.05 vs. control (Tukey’s post-hoc test). Results are presented as means ± SE. Target gene expression was normalized to that of the housekeeping gene, *Gapdh*, and the results are presented for each sample relative to the control. The expression levels of (**a**) *Cdkn2a*, (**b**) *Rbfox3*, (**c**) *Bdnf*, (**d**) *Ntrk2*, (**e**) *Fos*, (**f**) *100b*, (**g**) *Gfap*, (**h**) *Aif1*, (**i**) *Sirt1* are shown. (**j**) Table indicating the relative changes in expression of each gene in the control and extraction groups are indicated with arrows (YC vs. YE and AC vs. AE). *YC* young control group, *YE* young extraction group, *AC* aged control group, *AE* aged extraction group.

The expression of *Cdkn2a* (p16), which is related to the G1 arrest phase and is an indicator of senescence, was substantially increased in the YE3 and AE3 group mice compared with that in the YC3 and AC3 group mice, respectively (Fig. 3a). Furthermore, the expression of *Rbfox3*, a marker of neurons, was significantly decreased in mice of the AE3 group compared with that in mice of the AC3 group (Fig. 3b). The transcript-level expression of *Bdnf*, which is required for the development and maintenance of memory and learning, was significantly decreased in mice of the YE3 and AE3 group compared with that in mice of the YC3 and AC3 group, and this tendency was more pronounced in aged mice that had been subjected to molar extraction (Fig. 3c). However, the transcript-level expression of *Ntrk2*, a BDNF receptor, did not differ significantly between the extraction and non-extraction groups (Fig. 3d). Transcript-level expression of *Fos*, an indicator of functional neural activity, was also significantly decreased in mice of the AE3 group than in mice of the AC3 group (Fig. 3e). In young mice, the expression of *s100b* was clearly increased upon tooth extraction (Fig. 3f). Next, we examined the transcript-level expression of *Gfap*, an astrocyte marker (Fig. 3g). The expression of *Gfap* mRNA in young mice did not differ between the extraction group and the non-extraction group. However, a significant increase in the transcript-level expression of *Gfap* was observed in mice of the AE3 group compared with that in mice of the AC3 group (Fig. 3g). We also examined the transcript-level expression of *Aif1*, a microglia marker. We observed a significant increase in the expression of *Aif1* mRNA in the hippocampus of mice of the AE3 group compared with that in mice of the AC3 group (Fig. 3h). In addition, the transcript-level expression of *Sirt1*, a longevity gene^16^, was significantly decreased in both young and aged mice in the extraction group compared with that in the non-extraction group (Fig. 3i). The relative changes in expression of each of the above genes between the control (YC and AC) and extraction groups (YE and AE) are shown with arrows in a simplified tabular form (Fig. 3j).

**Figure 4:**
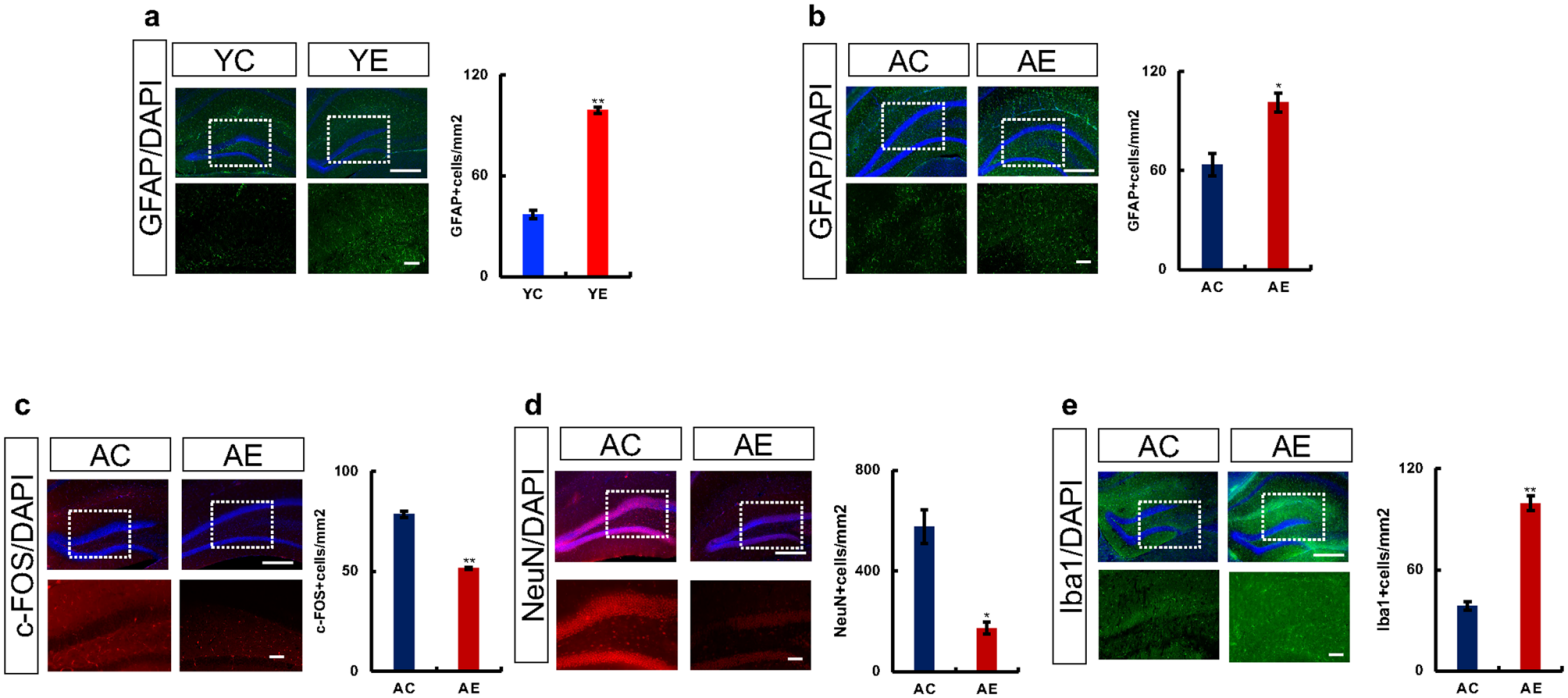
Effects of maxillary molar loss on protein expression in the hippocampus of mice (Immunostaining). Astrogliosis is induced in the hippocampus of mice with missing molars. The hippocampus and protein expression are shown. The square made by the dotted line is the CA1 region of the hippocampus, shown enlarged below. (**a**) Typical GFAP-positive cell area (mm^2^) and graphs showing the hippocampus of control and extraction groups of young mice (YC3 vs. YE3). (**b–e**) Typical staining images and graphs showing the hippocampus of control and extraction groups of aged mice (AC3 vs. AE3); (**b**) GFAP, (**c**) c-FOS, (**d**) NeuN, (**e**) Iba-1. The number of c-FOS- and NeuN-positive cells were increased, and c-FOS and NeuN positive cells were decreased upon tooth extraction. Scale bar: 100 μm.

Next, we examined the expression of these genes at the protein level by immunostaining brain tissues collected from the mice in both the tooth-extracted and non-extracted groups. In the hippocampus of mice from the YE3 and AE3 groups, the expression of *GFAP* was higher than that in mice from the YC3 and AC3 groups (Fig. 4a,b). In addition, a decrease in the number of NeuN-positive cells was observed in the hippocampus of mice from the AE3 group (Fig. 4d) as the expression of c-FOS decreased (Fig. 4c). An increase in the number of Iba1-positive cells was also observed in the hippocampus of mice from the AE3 group (Fig. 4e).

**Figure 5:**
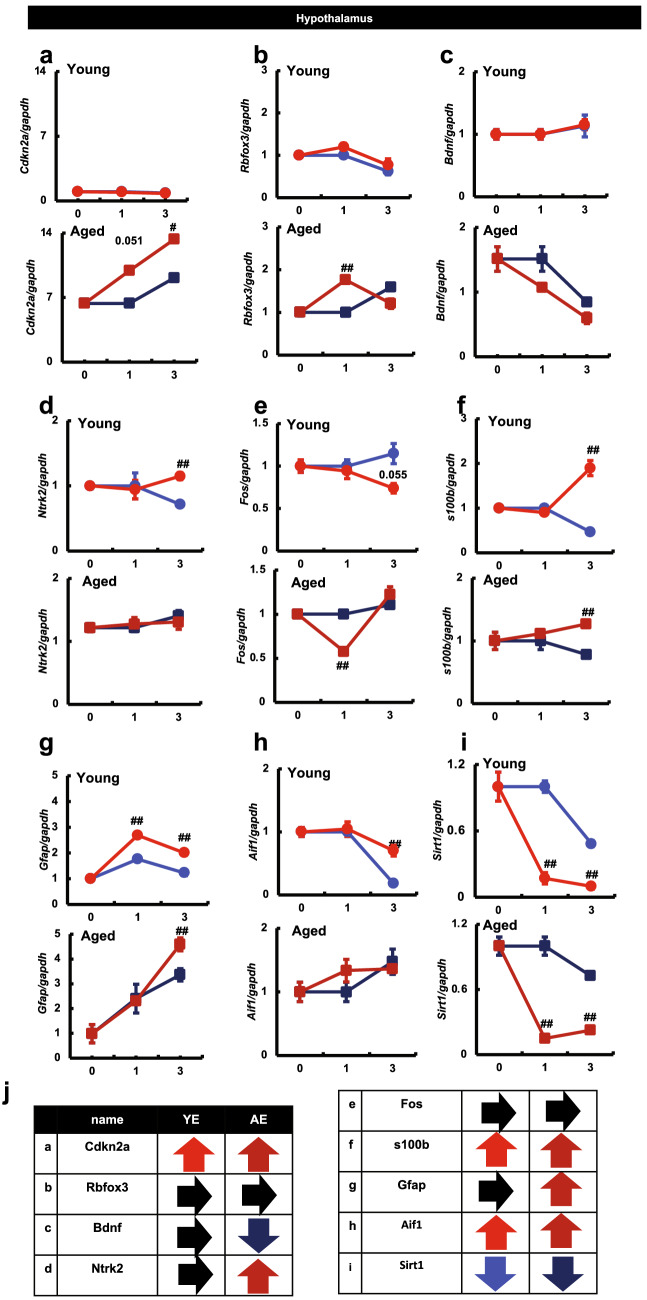
Effects of maxillary molar loss on molecular expression of hypothalamus in mice. The vertical axis is the expression level. The horizontal axis is the month, with YC1 or AC1 as 1. ^##^P < 0.01; ^#^P < 0.05 vs. control (Tukey’s post-hoc test). Results are presented as means ± SE. The vertical axis indicates the expression level. The horizontal axis indicates the month, with YC1 or AC1 as 1. ^##^P < 0.01; ^#^P < 0.05 vs. control (Tukey’s post-hoc test). Results are presented as means ± SE. Target gene expression was normalized to that of the housekeeping gene, *Gapdh*, and the results are presented for each sample relative to the control. The expression of (**a**) *Cdkn2a*, (**b**) *Rbfox3*, (**c**) *Bdnf*, (**d**) *Ntrk2*, (**e**) *Fos*, (**f**) *s100b*, (**g**) *Gfap*, (**h**) *Aif1*, and (**i**) *Sirt1* are shown. (**j**) The relative changes in expression of each gene (control group vs. extraction group) are shown in the table using arrows. *YC* young control group, *YE* young extraction group, *AC* aged control group, *AE* aged extraction group.

We investigated the mRNA expression of these molecules in the hypothalamus as in the hippocampus. The mRNA expression of *Cdkn2a* in the hypothalamus of young mice was lower than that in aged mice, and there was no difference in the mRNA expression of *Cdkn2a* between the extraction and non-extraction groups. However, in aged mice, the mRNA expression of *Cdkn2a* in the hypothalamus was enhanced over time in response to tooth extraction (Fig. 5a). In addition, no clear difference was observed between the extraction and non-extraction groups with respect to the transcript-level expression of *Rbfox3* (Fig. 5b). The mRNA expression of *Bdnf* in the hypothalamus decreased over time in aged mice, and this tendency was more pronounced in the tooth extraction group (Fig. 5c). However, the mRNA expression of *Ntrk2* did not differ between the extraction and non-extraction groups (Fig. 5d). No clear difference in transcript-level expression of *Fos* was observed between the extraction and non-extraction groups (Fig. 5e). The expression of *s100b* was markedly increased upon tooth extraction in both young and aged mice (Fig. 5f). The transcript-level expression of *Gfap* in the hypothalamus increased over time in aged mice, and the difference in expression between the extraction group and the non-extraction group became significant three months after tooth extraction (AE3) (Fig. 5g). The transcript-level expression of *Aif1* tended to increase over time in aged mice, but there was no difference between the extraction and non-extraction groups (Fig. 5h). In addition, *Sirt1* mRNA expression was significantly decreased in both young and aged mice in the extraction group compared with that in the mice of the non-extraction group (Fig. 5i). The relative changes in expression of each of the above genes between the control (YC and AC) and extraction groups (YE and AE) are shown with arrows in a simplified tabular form (Fig. 5j).

## Discussion

Astrogliosis, a central nervous system-specific response, occurs due to homeostasis in different brain regions^17,18^. Astrogliosis is characterized by the upregulation of GFAP and is observed in aging and neurodegenerative conditions, such as brain trauma, cerebral hemorrhage, and Alzheimer’s disease^19^. Meanwhile, astrocytes alter their morphology and proliferate in response to tissue damage^17^. This study is the first to demonstrate that tooth loss induces astrogliosis in the hypothalamus and hippocampus.

In this study, tooth extraction had little effect on mouse weight. This was likely because the anterior teeth of the mice were not treated, and the rodent-specific behavior of chewing with the anterior teeth was not impaired. We attempted to eliminate the effects of stress caused by tooth extraction by providing a one-month healing period after tooth extraction. As reported in the previous literature, healing of extraction sockets in rodents and replacement with new bone takes approximately 1–2 weeks^20,21^.

In our experiments, approximately 1 week after tooth extraction, the experimental group lost no more than 10% of their standard body weight. Several other studies have shown that tooth extraction in mice may result in a temporary weight loss immediately after tooth extraction, with no significant change in body weight thereafter^22,23^.

Regarding nutritional status after tooth extraction, it has been reported that amount of food intake did not change^24^, and in our experiments, there was no difference in blood glucose levels between the control and experimental groups. In our study, it is likely that the mice underwent a decrease in basal metabolism during long-term rearing as they aged, resulting in less body weight change.

Urinary corticosterone levels were unchanged in the young groups, but in aged mice, they increased in the extraction group. Previous studies have shown that plasma corticosterone levels in aged rats correlate significantly with hippocampal degeneration and impaired spatial learning ability^25^. Elevated plasma corticosterone levels are found only in aged rats with spatial dysfunction and not in rats with normal spatial memory^25^. Chewing can decrease the plasma corticosterone response in mice^26^. Onozuka et al. found that plasma corticosterone levels in the senescence-accelerated mouse-prone 8 (SAMP8) model 10 days after tooth extraction were significantly elevated in the test group compared with those in the control group^23^.

Kubo et al. measured the plasma corticosterone levels in SAMP8 8 months after tooth extraction^22^. They collected the sample at 20:00 h, at the beginning of the dark period, and we collected it at 10:00 h, during the light period. Since both studies assessed plasma corticosterone levels in SAMP8, their results cannot be directly compared with those from our study involving purely aged mice; however, it is evident that plasma corticosterone levels are elevated upon tooth loss^22^.

Mastication affects hippocampal function via glucocorticoids, end products of the hypothalamic–pituitary–adrenal (HPA) axis. Masticatory dysfunction affects the activation of the HPA axis^14,27^. In mice with no molars, the HPA negative feedback inhibition is impaired^28^. Long-term loss of molars in this study might have increased the corticosterone levels due to a chronic decrease in chewing volume, which modulated the HPA axis.

*Gfap* expression is upregulated with aging in astrocytes^29–31^. Here, real-time PCR and immunostaining showed that *Gfap* expression was significantly increased in the hippocampus and hypothalamus of mice that had been subjected to molar extraction compared with that in control mice. In contrast, no change was observed in *Gfap* expression in the hippocampus of young mice. This suggests that astrogliosis did not occur in young hippocampus. In the hippocampus of young mice, even if astrocytes are activated by pathological stimuli, the response quickly converges; this may be because there is no transition to gliosis. In the hypothalamus of young mice, *Gfap* expression was elevated upon tooth extraction (Fig. 5g). Reportedly, pathological stimuli are reflected more strongly in the hypothalamus^18^. However, in the hippocampus of young and aged mice, the response of astrocytes to pathological stimuli does not converge, and astrocytes may divide and proliferate, becoming reactive astrocytes, eventually resulting in gliosis^18^. This may have influenced the decline in learning and cognitive functions in aged mice with molar extraction^31^.

S100 is a protein expressed and released from astrocytes. It has been reported that the overexpression of S100 in the mouse brain causes memory impairment, and increased release of S100B from astrocytes may be involved in the memory impairment seen in the early stages of Alzheimer’s disease^32,33^. Iida et al. reported that the expression of S100a9 mRNA was decreased in the hippocampus of rats with missing teeth^34^. They also reported no difference in the expression of S100 between denture-wearing rats and controls. Although S100 expression alone cannot serve as a marker to understand the mechanisms underlying learning and memory, these findings suggest that S100 may influence memory and may also be affected by the presence or absence of occlusal support. S100 expression was significantly elevated in the hippocampus of the YE, AC, and AE group mice. However, S100B expression was significantly elevated in the hypothalamus of the YE and AE group mice. This suggests that S100B expression in the hippocampus increases with molar loss^34^.

In this study, changes were observed in the expression of various genes involved in brain function in the hippocampus and hypothalamus. *Bdnf* mRNA levels in the hypothalamus and hippocampus were decreased in aged mice with missing molars. The expression of *Ntrk2*, the receptor for *Bdnf*, was not affected by tooth extraction in either young or aged mice. However, the expression of *Fos* mRNA was decreased in the hippocampus of aged mice that had been subjected to tooth extraction. Behavioral experiments related to memory and anxiety revealed that the expression of c-FOS is upregulated in stimulus-responsive neurons^27^. Interestingly, prosthetic treatment in these mice resulted in a slight recovery of memory. It has also been shown that memory is stored in cells that are activated during learning and express c-FOS and Arc^35,36^. Therefore, the decreased expression of *Fos* mRNA in the hippocampus of aged mice with molar extraction might have been responsible for the decline in cognitive function in these mice.

*Aif1* is a marker of microglia. *Aif1* mRNA expression was upregulated in the hippocampus of both young and aged mice after tooth extraction. Microglia, as immunocompetent cells in the brain, maintain CNS homeostasis and regulate neurosynaptogenesis, pruning, and synaptic transmission. Activated microglia accumulate in the hippocampus of patients with neurodegenerative diseases such as Alzheimer’s disease and are thought to be involved in the pathogenesis of the disease.

Active microglia are broadly classified into M1 and M2 types, and the accumulation of M1-type microglia is thought to enhance inflammatory injury in the hippocampal tissue^37^. Here, we could not confirm that the accumulation of M1 macrophages or other inflammatory markers increased in the hippocampus^38^.

The expression of *Rbfox3*, a marker of neurons and specifically expressed in astrocytes, was markedly reduced in the hippocampus of aged mice with tooth extraction. It is possible that the neurons were shed by tooth extraction.

Here, we found that *Sirt1* expression in the hippocampus and hypothalamus was decreased in mice with missing maxillary first molars. *Sirt1*, a histone deacetylase^39^, enhances anti-aging and oxidative stress resistance, and its increased expression increases the lifespan of experimental animals^39^.

*Sirt1* is widely expressed in the brain and plays an important role in a variety of higher brain functions, including memory and learning formation, sleep, and circadian rhythm regulation. It has been shown that activation of *Sirt1* in the brain enhances synaptic plasticity, while loss of its activity impairs it^40^. In addition, brain *Sirt1* has been shown to exert central circadian control by activating the transcription of two major circadian regulators, BMAL1 and CLOCK^41^.

In the Y-maze behavioral experiment, we found that extraction of maxillary molars in aged mice significantly impaired learning and memory, a phenomenon that might have been influenced by decreased hippocampal *Sirt1* expression. In addition to the loss of circadian rhythms, deletion of *Bmal1* induces accelerated aging, cognitive decline, atrophy of skeletal muscle and other tissues, and a shortened lifespan in mice^42,43^. Mice with SCN-specific knockdown of *Bmal1* exhibit depressive behavior^44^. Young mice showed stronger gene expression after tooth extraction than aged mice, but there was no decline in cognitive function. In aged mice, the changes in gene expression were less dramatic, but the change in behavior was stronger. This may be due to the clock genes already being out of phase. Therefore, reduced expression of *Bmal1* may have affected the activation of nocturnal behavior. In addition, decreased *Bmal1* expression may be attributed to decreased *Sirt1* expression. Mice that were subjected to molar extraction exhibited an increased number of arm entries in the Y-maze (Fig. 2c,d), which may have been due to anxiety. The poor performance in the Y-maze task may be related to the loss of the first maxillary molar. Furthermore, it may be affected by the decreased expression of *Bmal1*, *Fos*, and *Bdnf* in the hippocampus. It is plausible that the tooth-extracted mice were anxious during the first month, but by the third month, they had acclimated to the condition.

Decreased masticatory function owing to tooth loss has been shown to decrease cerebral blood flow and degenerate mastication-related neocortical areas^45,46^. Mastication enhances cerebral functions, such as spatial memory, increases alertness and attention, and improves cognitive abilities^47–49^. Molar extractions and the resulting occlusal imbalance have been associated with abnormal pressure on the palatal system, increased plasma corticosterone levels, degeneration of hippocampal neurons and glial cells, and decreased spatial memory^17,28^. Masticatory activity influences cell proliferation and neurogenesis in the adult dentate gyrus^49^. Taken together, these findings strongly suggest that the loss of molars results in the development of central nervous system disorders. A possible mechanism by which the loss of masticatory function affects brain functions is that afferent signals from the trigeminal nerve affect the hippocampus. Intraoral sensation from mastication is captured by the sensory branches of the trigeminal nerve in the periodontal ligament and muscle spindles of the masseter muscle and propagated to the mesencephalic trigeminal nucleus. Goto et al. confirmed that molar extraction worsened cognitive function in Alzheimer’s disease model mice, and they found that the number of neurons decreased in the mesencephalic trigeminal nucleus, adjacent nucleus accumbens, and the hippocampus of the same mice^11^. In our study, we used male mice. However, age and sex can impact metabolism in both humans and rodents^50^. As the nucleus accumbens is adjacent to the mesencephalic trigeminal nucleus, some interaction might exist between these structures. The nucleus accumbens also projects axons into many regions and plays an important role as a central noradrenaline-producing apparatus, and damage to the nucleus accumbens is known to directly affect hippocampal function^51^. In summary, molar loss may directly affect the trigeminal nucleus of the mesencephalon, which is propagated to the pontine nucleus and then to the hippocampus, thereby affecting cognitive learning functions. On the dorsal side of the main sensory nucleus of the trigeminal nerve, some neurons exhibit rhythmic activity synchronized with mastication, and the interaction of neurons and astrocytes in this area maintains the functions of this area^50^. Herein, molar-deficient mice developed astrogliosis as a result of decreased sensation transmitted from the periodontal sensory receptors of (at least) the missing tooth to the trigeminal nerve. Astrogliosis is also associated with cognitive function and anxious behavior^18^. Therefore, suppression of the HPA axis in response to tooth loss resulted in increased cortisol levels, thereby downregulating the expression of *Bdnf*, which in turn resulted in increased *Gfap* expression, ultimately inducing astrogliosis. Astrogliosis may have resulted in a decrease in the expression of hypothalamic *Bmal1*, thereby inducing a loss of circadian rhythm via the suprachiasmatic nucleus, increased nocturnal behavior, and decreased cognitive function^53^. To the best of our knowledge, the formation of astrogliosis in the hippocampus and hypothalamus of the brain due to tooth loss is being reported for the first time in this report. Our results clarify the relationship between tooth loss and brain inflammation. In this study, we examined the brain at 1 and 3 months after tooth extraction in mice. This translates to a long period of time (~ 2–7 years) in humans, and prosthetic treatment is usually necessary. It is likely that in the first month after tooth extraction, the mice were still getting used to their condition, and only a few behavioral and genetic changes were observed; however, by the third month, the mice strongly exhibited symptoms of anxiety. This study is the first to explore the long-term effects of tooth loss on the brain in purely aged mice. In the future, we would like to study, in more depth, the long-term effects of tooth extraction on other body parts.

This study revealed that tooth loss not only interferes with masticatory function but also induces changes in brain function, as the decrease in masticatory-related stimuli from various sensory receptors, such as periodontal ligament receptors, is projected onto various regions of the brain^54^. Additionally, these changes may be attributed to the underlying alterations in gene expression in the hippocampus and hypothalamus. Although missing teeth cannot be restored, restoring masticatory function with prosthetics may increase mastication-related stimulation from the periphery to the center and suppress the degeneration of neurotransmission circuits and brain tissue. Thus, dental prostheses may be important for maintaining brain function.

## Methods

### Animals

We obtained 6-week-old male C57BL/6N mice from Chubu Kagaku Shizai Co., Ltd. Japan (Aichi, Japan). We also obtained 18-month-old male C57BL/6N mice from the aging farm at NCGG. The mice were housed under a 12 h light/dark cycle, with lights off from 19:00 to 07:00. A CLEA Rodent Diet CE-2 (CLEA Japan Inc; Tokyo, Japan), which is a GLP-compliant, standard rodent diet consisting mainly of vegetable protein (soybean meal) with a proper balance of animal protein was given. All groups were allowed to feed and drink water freely. They were randomly divided into the YC group (Young mice, intact upper first molar teeth, solid diet feeding, n = 18), AC group (Aged mice, intact upper first molar teeth, solid diet feeding, n = 19), YE group (Young mice, extracted upper molar teeth, solid diet feeding, n = 25), and AE group (Aged mice, extracted upper molar teeth, solid diet feeding, n = 18). In the YE and AE group mice, the maxillary first molar teeth on both sides were extracted at the age of 8–10 weeks (YE0) or 18 months (AE0), under general anesthesia with pentobarbital sodium (35 mg/kg, i.p. Somnopentyl; Kyoritsu Seiyaku Corporation, Tokyo, Japan). Mice were fixed on a special fixator (NAIGAI-CFK-2S, AS ONE Corporation, Osaka, Japan) for small animal experiments. In the extraction group, maxillary bilateral first molars were extracted by dislocating them with a dental explorer. After tooth extraction, an anesthetic antagonist was administered and no analgesic was given. These procedures took up to 30 min. No analgesics or anti-inflammatory medications were administered postoperatively. In contrast, the YC and AC groups received only anesthesia.

Each mouse was subjected to behavioral experiments and euthanized one and three months after molar extraction. The number of mice used per group was as follows: YC0 (n = 6), YC1 (n = 6), YC3 (n = 6), YE0 (n = 8), YE1 (n = 8), YE3 (n = 9), AC0 (n = 6), AC1 (n = 6), AC3 (n = 7), AE0 (n = 6), AE1 (n = 6), and AE3 (n = 6). YE and AE mice were also sacrificed for the collection of brain expression data, and were sacrificed 10 days (E0), 1 month (E1), and 3 months (E3) following extraction.

All the experiments involving animals were approved by the animal care and use committee at the National Center for Geriatrics and Gerontology (NCGG) in Japan (approval number 2-23). All the experiments were performed in compliance with the Guide for the Care and Use of Laboratory Animals published by the United States National Institutes of Health (NIH Publication, 8th Edition, 2011). The study was carried out in compliance with the ARRIVE guidelines.

### Body weight and general health

After 7 days habituation, all mice were weighed on a laboratory scale accurate to 0.01 g and inspected for signs of ill health, including wounds and poor body condition. Body weight was measured every week.

### Blood glucose level measurement

Blood glucose levels in mice were measured with LaboGluco (FORA Care Japan Co. Ltd., Tokyo, Japan), using blood collected from the heart at the time of sacrifice.

### Urine corticosterone

Although corticosterone can be measured in the serum or feces, we chose to measure urinary corticosterone levels because this method is less invasive and less subject to handling-associated stress than the collection of serum^55^. Further, unlike obtaining feces, it is not subject to microbial metabolism and can be controlled for differences in output or concentration, as it is a molecule filtered at a constant rate by the kidneys^56^. One month after tooth extraction, we noninvasively collected a urine sample from each mouse between 10:00 and 12:00 h. This schedule was maintained consistently to control for circadian variations in baseline corticosterone levels^57^. Urination was stimulated by transferring the mice individually to clean plastic containers and stimulating the base of their tail. Urine was transferred to a sealed microcentrifuge tube by using a 1-mL syringe and stored at − 80 °C. After storage, samples were thawed, diluted 26-fold with sterile water and tested for corticosterone using plate ELISA (ARK Checker CORT EIA, ARK Resource, Kumamoto, Japan) as per the manufacturer’s instructions.

### Amount of drinking water and feed intake

Daily water and feed intake were calculated by placing each mouse in a 24-h activity measuring device (Time HC8_Single, O’Hara) after 4 h of acclimation.

### Behavioral testing

#### Y-maze test

Spontaneous alternation behavior was assessed in a gray, acrylic Y-shaped maze, which consists of three arms (60 cm long × 60 cm wide × 25 cm deep, YM-3002, O’Hara & Co. Ltd., Tokyo, Japan) projecting from each side of a central equilateral triangle. The Y-maze test was performed as specified by Wahl et al*.*^58^. The learning rate was measured based on the number of sessions required to achieve this criterion. A mouse was placed in one arm (no. 1) where it had three options as its first choice: staying in arm 1, moving into arm 2, or moving into arm 3. An alternation was considered as correct if the mouse visited a new arm and did not return to the two previously visited arms. The ratio of correct alternations to the number of visits during a 10-min observation period was calculated as the frequency of alternation^59^. A frequency > 50% indicated spontaneous alternation. The test was repeated, and the number of correct responses were taken as a measure of memory. The dependent variable was percentage alternation, calculated as^60^:

$${\text{Alteration }}\left( \% \right)\, = \,\left( {{\text{number of alternations}}\, \div \,\left[ {{\text{total entries}}\, - \,{2}} \right]} \right)\, \times \,{1}00.$$

#### Motor skill learning test

A rotarod machine with automatic timers and falling sensors (MK-660D, Muromachi-Kikai, Tokyo, Japan) was used. We referred to the RIKEN (Saitama, Japan) BioResource Center, Standard Operating Procedures (BRC’s SOP). A mouse was placed on a 9-cm-diameter drum. The surface of the drum was covered with hard chloroethylene, which prevents gripping on the surface. We set the unit to accelerate from 4 to 40 rpm in 300 s. After the drop, the animal was allowed to rest for 20 min and then placed back on the drum a maximum of two times in one session. To evaluate long-term memory, the test was repeated once daily for two consecutive days. The latency until the mouse fell was recorded automatically on the rod each day.

### Real-time PCR

Total RNA was isolated from the hypothalamus and hippocampus using the Nucleospin RNA kit (cat no. U0955C; Takara Bio Inc., Shiga, Japan) as per the manufacturer’s instructions. The total RNA concentration was corrected to 100 ng/μL with a NanoDrop 2000 spectrophotometer (Thermo-Fisher Scientific, Waltham, MA, USA). First-strand cDNA synthesis was performed using the ReverTra Ace-α Kit (TOYOBO, Osaka, Japan). Real-time PCR was performed using a FastStart Essential DNA Green Master (Roche, Mannheim, Germany) according to the manufacturer’s protocol. Real-time PCR was performed on a LightCycler 96 System (Roche, Mannheim, Germany). The primer sequences are presented in Table 1. Target gene expression was normalized to that of the housekeeping gene *Gapdh,* and the results are presented for each sample relative to the control^14,60^. These experiments were performed in triplicate for each condition. The values are presented as the fold change between samples using the 2^−ΔΔCt^ method^61^.

**Table 1:** Sequences of primers used for real-time PCR.

Name	Forward	Reverse
Mouse Glut1	TCAACACGGCCTTCACTG	CACGATGCTCAGATAGGACATC
Mouse Bmal1	TGCCACCAATCCATACACAG	TTCCCTCGGTCACATCCTAC
Mouse Drd2	ATCTCTTGCCCACTGCTCTTTGGA	ATAGACCAGCAGGGTGACGATGAA
Mouse Cdkn2a (p16)	CGTACCCCGATTCAGGTGAT	TTGAGCAGAAGAGCTGCTACG
Mouse Sirt1	ATGACGCTGTGGCAGATTGTT	CCGCAAGGCGAGCATAGAT
Mouse Gfap	TCCTGGAACAGCAAAACAAG	CAGCCTCAGGTTGGTTTCAT
Mouse Bdnf	TGCAGGGGCATAGACAAAAGG	CTTATGAATCGCCAGCCAATTCTC
Mouse Ntrk2 (TrkB)	AAGGACTTTCATCGGGAAGCTG	TCGCCCTCCACACAGACAC
Mouse Fos (c-fos)	GGGGACAGCCTTTCCTACTA	CTGTCACCGTGGGGATAAAG
Mouse Aif1 (Iba1)	CTTTTGGACTGCTGAAGGC	GTTTCTCCAGCATTCGCTTC
Mouse Rbfox3 (NeuN)	CACCACTCTCTTGTCCGTTTGC	GGCTGAGCATATCTGTAAGCTGC
S100b	CCCTCATTGATGTCTTCCACC-	TCTCCATCACTTTGTCCACC
Mouse GAPDH	AACCTGCCAAGTATGATGA	GGAGTTGCTGTTGAAGTC

### Immunohistochemistry

We analyzed at least three mice brains per group for immunohistochemistry, and a minimum of 10 sections were quantified per mouse. Brains were excised from the experimental mice and immersed for 24 h in PBS. Serial coronal section (30 μm) obtained at the level of the bregma, (1.5–2 mm posterior ± 1.2–1.3 mm mediolateral) were processed for immunohistochemistry using VT1200S (Leica Microsystems, Wetzlar, Germany). We then blocked nonspecific binding for 1 h using PBS containing 1% Triton X-100 and 10% normal donkey serum (S30–100 ml, Merck). After three washes, brain slices were incubated overnight (with shaking at 4 °C) with the corresponding primary antibodies diluted in PBS, i.e., anti-GFAP (1:1000; ab4674, Abcam, Cambridge, UK), anti-cFOS (1:1000; MCA-2H2, EnCor Biotechnology Inc, Florida, USA), and anti-Iba1 (1:200; ab178846, Abcam), anti-F (1:1000; ab104224, Abcam). After incubation with the primary antibody, the tissue slices were washed thrice with PBS and then incubated with subtype-specific fluorescent secondary antibodies, i.e., goat anti-chicken IgY (H + L) antibody (1:500, A-11039, Invitrogen), donkey anti-mouse anti-rabbit IgG (H + L), Alexa Fluor 555 (1:200; ab 150110, Abcam) for 1 h at 25 °C with shaking. Controls were incubated in the absence of the primary antibody. Sections were observed using Keyence BZ-X800 (KEYENCE Co., Osaka, Japan). The number of positive cells in the CA1 region was counted using Image J software v1.52a (NIH, USA), and quantitative analysis of each immunolabeled area was performed by three individuals.

### Statistical analysis

All values are presented as mean values ± standard errors of the mean (SEM). T-tests were used for evaluating the control and experimental groups. When significant effects were detected, post-hoc analyses were performed using Tukey’s post-hoc test. P values < 0.05 were considered significant. All statistical analyses were performed using EZR^62^, a modified version of R commander that adds statistical functions frequently used in biostatistics.

## Data Availability

Source data are available as a Source Data file. All other data are available from the corresponding authors upon reasonable request.
