# Author Correction: Molar loss induces hypothalamic and hippocampal astrogliosis in aged mice

**DOI:** 10.1038/s41598-022-17094-2

**Published:** 2022-07-25

**Authors:** Masae Furukawa, Hirobumi Tada, Jingshu Wang, Mitsuyoshi Yamada, Mie Kurosawa, Akiko Satoh, Noboru Ogiso, Yosuke Shikama, Kenji Matsushita

**Affiliations:** 1 Department of Oral Disease Research, Geroscience Research Center, National Center for Geriatrics and Gerontology, Obu, Japan; 2 Department of Nutrition, Faculty of Wellness, Shigakkan University, Obu, Japan; 3 Department of Infammation and Immunosenescence, Geroscience Research Center, National Center for Geriatrics and Gerontology, Obu, Japan; 4 Department of Operative Dentistry, School of Dentistry, Aichi Gakuin University, Nagoya, Japan; 5 Department of Integrative Physiology, Geroscience Research Center, National Center for Geriatrics and Gerontology, Obu, Japan; 6 Department of Integrative Physiology, Institute of Development, Aging, and Cancer, Tohoku University, Sendai, Japan; 7 Department of Laboratory of Experimental Animals, National Center for Geriatrics and Gerontology, Obu, Japan

Correction to: *Scientific Reports* 10.1038/s41598-022-10321-w, published online 18 April 2022

The original version of this Article contained an error in Figure 4B, where the labels AC and AE were interchanged in the GFAP graph.

The original Figure 4 and accompanying legend appear below.

**Figure 4:**
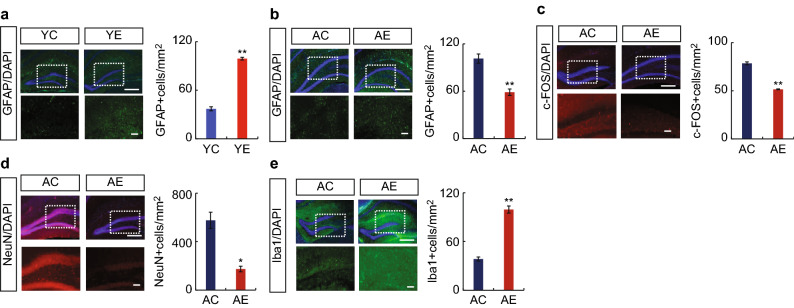
Effects of maxillary molar loss on protein expression in the hippocampus of mice (Immunostaining). Astrogliosis is induced in the hippocampus of mice with missing molars. The hippocampus and protein expression are shown. The square made by the dotted line is the CA1 region of the hippocampus, shown enlarged below. (**a**) Typical GFAP-positive cell area (mm^2^) and graphs showing the hippocampus of control and extraction groups of young mice (YC3 vs. YE3). (**b–e**) Typical staining images and graphs showing the hippocampus of control and extraction groups of aged mice (AC3 vs. AE3); (**b**) GFAP, (**c**) c-FOS, (**d**) NeuN, (**e**) Iba-1. The number of c-FOS- and NeuN-positive cells were increased, and c-FOS and NeuN positive cells were decreased upon tooth extraction. Scale bar: 100 μm.

The original Article has been corrected.

